# Anti-staphylococcal activity resulting from epithelial lining fluid (ELF) concentrations of amikacin inhale administered via the pulmonary drug delivery system

**DOI:** 10.1186/s12941-017-0178-0

**Published:** 2017-01-17

**Authors:** Islam M. Ghazi, Mordechai Grupper, David P. Nicolau

**Affiliations:** 1 Center for Anti-Infective Research and Development, Hartford Hospital, 80 Seymour Street, Hartford, CT 06102 USA; 2 Division of Infectious Diseases, Hartford Hospital, Hartford, CT USA

**Keywords:** Pharmacodynamics, Inhaled amikacin, *S. aureus*

## Abstract

**Background:**

Amikacin inhale (BAY41-6551), a unique drug—device combination of a specially formulated drug solution and a pulmonary drug delivery system device (AMK-I) is currently under phase III study as an adjunctive therapy to IV antibiotics for the treatment of Gram-negative pneumonia in mechanically ventilated patients. While the epidemiology of nosocomial pneumonia is predominated by Gram-negative pathogens such as *Pseudomonas aeruginosa* and the Enterobacteriaceae, *Staphylococcus aureus* is increasingly recognized as a pathogen of concern for these pulmonary based infections. Since the aminoglycosides are historically quite active against *S. aureus* the use of adjunctive AMK-I may enhance bacterial eradication. Herein, we aimed to characterize the in vitro pharmacodynamic (PD) profile of human-simulated ELF exposures of AMK-I against both methicillin-sensitive (MSSA) and -resistant (MRSA) *S. aureus*.

**Methods:**

An in vitro model was used to simulate the resultant ELF pharmacokinetic profile of amikacin after the administration of AMK-I 400 mg q12h. The antibacterial activity of this regimen was tested against 7 *S. aureus* isolates that display MIC profiles encountered clinically (4 MRSA; MIC range 4–64, 3 MSSA; MIC range 8–16 mg/L). Experiments were conducted over 24 h and samples were taken throughout this period to assess the bacterial density in both control and treatments.

**Results:**

The mean ± SD inoculum 0 h bacterial density was 6.4 ± 0.09 which increased to 8.6 ± 0.19 log_10_ CFU/mL in the control models by the end of 24 h experiments. Simulated ELF concentrations of AMK-I resulted in a rapid, 5 log_10_ declined in CFU over the initial 12 h for all MRSA and MSSA isolates. After 12 h, all bacterial counts remained below the limit of detection (LOD, 1.7 log_10_ CFU/mL) and no regrowth was evident at the end of the study.

**Conclusion:**

AMK-I produced an ELF exposure profile that was rapidly bactericidal against *S. aureus* displaying typical MICs to amikacin irrespective of their phenotypic profile to methicillin. While the Gram-negative organisms are the target pathogens for AMK-I in the ongoing clinical trials, these data suggest that this adjunctive regimen may also have the potential to eradicate both MSSA and MRSA from lower airway which needs to be further evaluated in randomized-controlled clinical trials.

## Background

It has been long recognized that amikacin displayed potent activity against *Staphylococcus aureus* including methicillin-resistant (MRSA); however, due to availability of other less toxic agents, amikacin has been historically reserved for Gram-negative pathogens [1, 2]. Although Gram-negative organisms are prevalent in ventilator associated pneumonia (VAP), *S. aureus* phenotypes (MSSA and MRSA) are increasingly abundant and may account for up to 28% of infecting pathogens [3].

While parenteral amikacin has been useful for the treatment of variety of infections, including VAP, the emergence of multidrug resistant pathogens and the persistent potency of amikacin have re-invigorated interest in the compound. As a result of the challenge of delivering sufficiently high concentrations of aminoglycosides to the lungs for the treatment of VAP, a novel inhaled amikacin formulation was developed. Amikacin inhale (BAY41-6551) is a drug-devise combination currently being assessed for the management of Gram-negative pneumonia in the intubated patients. The pulmonary drug delivery system (PDDS) nebulizer can be attached to the standard mechanical ventilation equipment or to portable handheld unit delivering approximately 50–70% of the nominal dose to the lower airways [4]. Previously we have shown that the resultant high local bronchopulmonary exposure profile of amikacin achieved with this administration technique was able to eradicate *Enterobacteriaceae* and *Pseudomonas aeruginosa* despite elevated MICs as currently defined by clinical laboratory criteria [5]. In addition to the high achievable amikacin ELF concentrations after inhalation another attribute of this pulmonary delivery technique is the low systemic exposure [6].

As a result of the prevalence of polymicrobial infections with *S. aureus* in VAP, we sought to investigate the anti-staphylococcal activity of humanized ELF exposure of amikacin inhale delivered via PDDS.

## Methods

Due to the limited availability of commercially prepared amikacin intravenous solution, pharmaceutical grade amikacin powder (Medisca, Plattsburg, NY, USA) was acquired from our institutions’ pharmacy for use in in vitro pharmacodynamic model. Prior to the conduct of the pharmacodynamic studies, an initial run was undertaken in duplicate to confirm the equal biological potency of amikacin solution when prepared from the powder and commercially available vials. Standard analytical grade amikacin was purchased from Sigma-Aldrich (St. Louis, MO, USA) for MIC testing as per Clinical Laboratory Standards Institute (CLSI) recommendations. MIC studies were performed in triplicate by broth micro dilution per CLSI [7] methodology and the modal value reported. *S. aureus* isolates (n = 24) from a previous surveillance study [8] were screened and 7 were included (3 MSSA; MIC 8–16 mg/L and 4 MRSA; MIC 4–64 mg/L) to cover 99% of MIC range encountered clinically [9].

The mean steady-state ELF concentrations of amikacin 400 mg q12h delivered via PDDS [10], were simulated in the in vitro pharmacodynamic model with target peak and trough concentration of 5250 and 507 mg/L, respectively, resulting in an area under the curve (AUC_0−12_) of 17,940 mg h/L. A simulated dynamic in vitro model was used for all experiments as previously described [11]. Briefly, each experiment consisted of two treatment models and one growth control model running simultaneously for each isolate. For optimal temperature control, the models were placed in a 37 °C water bath operated by temperature and circulation controller (PolyScience, Niles, IL, USA).

A starting (0 h) inoculum in each model was targeted to be ~10^6^ CFU/mL. Each model was filled with cation adjusted Mueller–Hinton Broth (CAMHB) (Becton, Dickinson and Company, Sparks, MD) and inoculated 30 min before starting dosing, then amikacin was administered at simulated exposures (0 h). A peristaltic pump (Masterflex L/S Model 7524-40; Cole-Parmer Instrument Company, USA) was utilized to simulate half-life in ELF. From a pharmacokinetic standpoint, the model consists of 300 mL glass containers representing the volume of distribution, the drug is bolused to achieve the desired peak and pumps deliver fresh CAMHB to the glass containers representing drug clearance to mimic calculated half-life. From a pharmacodynamics standpoint, samples were obtained from each model at pre-defined time points to assess changes in bacterial density relative to drug-free control model. Aliquots of each diluted sample were plated on Trypticase soy agar plates with 5% sheep blood (BAP, BD Biosciences, Sparks, MD) and incubated at 37 °C for 16–20 h for quantitative culture. To eliminate the interference of antibiotic carry over, 2 safeguards were applied; samples were serially diluted and if any area of inhibited growth had been observed on the plate, it was excluded from the study. As a measure of the antibacterial activity over the 24 h, log_10_ CFU/mL of test isolate was plotted versus time. The lower limit of detection for bacterial density was 1.7 log_10_ CFU/mL. To ensure target exposures in the model, amikacin concentrations were sampled at pre-defined time points, and assayed by Quest Diagnostics (Chantilly, VA, USA) using a commercially available kit (Siemens Healthcare Diagnostics, Deerfield, IL, USA).

## Results

A comparison of the amikacin solutions prepared from the powder and commercially available vials revealed a similar time-killing profile thus proving the interchangeability of the amikacin sources. As a result of the availability of sufficient quantities of amikacin powder, this preparation was utilized in all in vitro modeling experimentation.

**Fig. 1:**
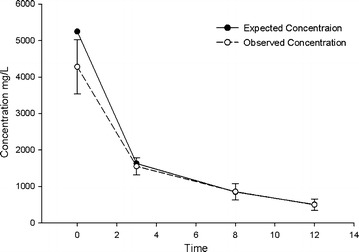
Expected and observed amikacin epithelial lining fluid profiles

**Fig. 2:**
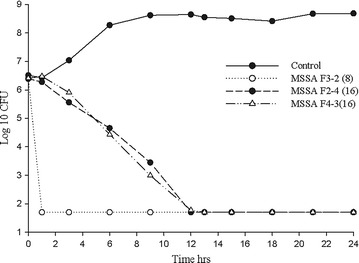
Mean bacterial density of MSSA isolates tested (amikacin MIC, mg/L) in simulated epithelial lining fluid concentrations of inhaled amikacin

**Fig. 3:**
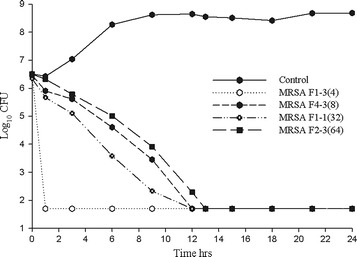
Mean bacterial density of MRSA isolates tested (amikacin MIC, mg/L) in simulated epithelial lining fluid concentrations of inhaled amikacin

The observed pharmacokinetic parameters (mean ± SD) for amikacin derived from the 14 individual experiments (peak 4282 ± 743 mg/L, trough 498 ± 154 mg/L, AUC_0−12_ 16512 ± 2229 mg h/L) were similar to the targeted values (Fig. 1). The average bacterial density of the starting inoculum was 6.4 ± 0.09 log_10_ CFU/mL, increasing to 8.6 ± 0.19 4 log_10_ CFU/mL in control models by the end of the 24 h period. Figures 2 and 3 show the mean bacterial densities over the 24 h exposure period for MSSA and MRSA isolates respectively. Amikacin simulated concentration achieved maximal killing effect within 12 h, and sustained bactericidal activity at the lower limit of detection (1.7 log_10_ CFU/mL) over 24 h. While the overall extent of kill was similar for both MSSA and MRSA isolates, an initially faster rate of kill was observed for some isolates with MICs of 4–8 mg/L.

## Discussion

The current study demonstrates, using an in vitro model, that pulmonary amikacin exposures achieved by inhalation via PDDS are highly active against *S. aureus* isolates irrespective of methicillin resistance phenotype, up to a MIC of 64 mg/L which encompasses 99% of clinical isolates [9].

From a pharmacodynamic standpoint, the initial pronounced killing and sustained effect noted, fits the aminoglycosides’ well established characteristics of rapid initial binding to the bacterial cell and prolonged persistent bactericidal activity [12]. Moreover, our observed killing profile is consistent with the established pharmacodynamic targets of the aminoglycosides as our calculated parameters of AUC_0−12_/MIC and peak/MIC for these organisms were well above that required for antibacterial efficacy [13].

Despite the lack of clinical data with amikacin, studies have demonstrated the utility of other aminoglycosides against infections caused by *S. aureus* as in case of gentamicin that successfully treated several infection sites [14]. Recently arbekacin was found to be a safe and effective alternative to vancomycin to treat *S. aureus* infections [15]. These data prove the efficacy of aminoglycosides against *S. aureus* but monotherapy is not a commonplace.

One potential limitation of our study is the absence of surfactants in the model. While it has been shown that lung surfactant affects the activity of antibiotics due to drug binding to surfactant proteins or phospholipids, the aminoglycosides are lowly protein bound and poorly charged agents and thus are not expected to bind to this biological matrix. Moreover, since inhaled amikacin is being investigated in clinical trials, we aimed to demonstrate the microbiologic activity resulting from the achievable ELF concentrations of the compound against *S. aureus* to provide insights regarding its antibacterial properties in the lower airway [16–18].

An additional limitation of the current study is the use of a PK model that was derived by conducting simulation with PK data of mechanically ventilated patients (n = 28) with lower respiratory tract infections who were administered amikacin through PDDS [6]. Although the simulated model was not validated in a clinical study, we believe that the patient population and thus the derived amikacin ELF profile is sufficiently robust to support its use in our preclinical evaluation against *S. aureus*. Nonetheless, the results of preclinical studies should be interpreted with caution and substantiated by clinical evidence before the intervention of interest is integrated into the management of the infected patients.

## Conclusions

While the notion of amikacin antistaphylococcal activity is recognized, the current study points out that the exquisitely high amikacin concentrations achieved locally in the lungs through inhalation coupled with the compound’s inherent bactericidal activity provide extensive killing of *S. aureus* inclusive of those isolates with MICs at the top end of the distribution (i.e., MIC 64 mg/L). Recognizing that clinical trial data are required to define the role of amikacin inhale for the management of VAP due to *S. aureus*, the current data set provides new insights into the potential utility of this novel therapeutic approach for this increasingly prevalent pathogen in the setting of nosocomially acquired bronchopulmonary infections.

## Abbreviations

AMK-I: amikacin inhale
AUC_0-12_: area under the curve from 0 to 12 h
CAMHB: cation adjusted Mueller–Hinton broth
CFU: colony forming unit
CLSI: Clinical and Laboratory Standards Institute
ELF: epithelial lining fluid
IV: intravenous
PK: pharmacokinetics
PD: pharmacodynamics
PDDS: pulmonary drug delivery system
MIC: minimum inhibitory concentration
MRSA: methicillin-resistant *Staphylococcus aureus*
MSSA: methicillin-sensitive *Staphylococcus aureus*
SD: standard deviation
VAP: ventilator associated pneumonia

## References

[1] Marks MI (1975). In vitro antibacterial activity of amikacin, a new aminoglycoside, against clinical bacterial isolates from children. J Clin Pharmacol.

[2] Del Favero A, Menichetti F, Guerciolini R, Bucaneve G, Baldelli F, Aversa F (1987). Prospective randomized clinical trial of teicoplanin for empiric combined antibiotic therapy in febrile, granulocytopenic acute leukemia patients. Antimicrob Agents Chemother.

[3] Weber DJ, Rutala WA, Sickbert-Bennett EE, Samsa GP, Brown V, Niederman MS (2007). Microbiology of ventilator-associated pneumonia compared with that of hospital-acquired pneumonia. Infect Control Hosp Epidemiol.

[4] Dhand R, Sohal H (2008). Pulmonary drug delivery system for inhalation therapy in mechanically ventilated patients. Expert Rev Med Devices.

[5] So W, Crandon JL, Hamada Y, Nicolau DP (2016). Antibacterial activity of achievable epithelial lining fluid exposures of amikacin inhale with or without meropenem. J Antimicrob Chemother.

[6] Luyt CE, Clavel M, Guntupalli K, Johannigman J, Kennedy JI, Wood C (2016). Pharmacokinetics and lung delivery of PDDS-aerosolized amikacin (NKTR-061) in intubated and mechanically ventilated patients with nosocomial pneumonia. Crit Care.

[7] Clinical and Laboratory Standards Institute (2015). Performance standards for antimicrobial susceptibility testing: twenty-fourth informational supplement [document M100-S25].

[8] Housman ST, Sutherland CA, Nicolau DP (2014). Pharmacodynamic profile of commonly utilised parenteral therapies against meticillin-susceptible and meticillin-resistant Staphylococcus aureus collected from US hospitals. Int J Antimicrob Agents.

[9] Sader HS, Rhomberg PR, Farrell DJ, Jones RN (2015). Arbekacin activity against contemporary clinical bacteria isolated from patients hospitalized with pneumonia. Antimicrob Agents Chemother.

[10] Stass H, Willmann S, Wendl T. Risk assessment for amikacin inhale in ICU patients using whole-body physiologically based PK-models. Poster 926. Society of critical care medicine (SCCM) 43rd critical care congress. San Francisco; 2014.

[11] Blaser J, Zinner SH (1987). In vitro models for the study of antibiotics activities. Prog Drug Res.

[12] Zhanel G, Hoban D, Harding G (1991). The postantibiotic effect: a review of in vitro and in vivo data. DICP Ann Pharmacother..

[13] Kashuba AD, Bertino JS, Nafziger AN (1998). Dosing of aminoglycosides to rapidly attain pharmacodynamic goals and hasten therapeutic response by using individualized pharmacokinetic monitoring of patients with pneumonia caused by Gram-negative organisms. Antimicrob Agents Chemother.

[14] Richards F, McCall C, Cox C (1971). Gentamicin treatment of staphylococcal infections. JAMA.

[15] Hwang JH, Lee JH, Moon MK, Kim JS, Won KS, Lee CS (2013). The efficacy and safety of arbekacin and vancomycin for the treatment in skin and soft tissue MRSA infection: preliminary study. Infect Chemother..

[16] Schwameis R, Erdogan-Yildirim Z, Manafi M, Zeitlinger MA, Strommer S, Sauermann R (2013). Effect of pulmonary surfactant on antimicrobial activity in vitro. Antimicrob Agents Chemother.

[17] Silverman JA, Mortin LI, Vanpraagh AD, Li T, Alder J (2005). Inhibition of daptomycin by pulmonary surfactant: in vitro modeling and clinical impact. J Infect Dis.

[18] van ‘t Veen A, Mouton JW, Gommers D, Kluytmans JA, Dekkers P, Lachmann B (1995). Influence of pulmonary surfactant on in vitro bactericidal activities of amoxicillin, ceftazidime, and tobramycin. Antimicrob Agents Chemother.

